# Understanding Infection-Induced Thrombosis: Lessons Learned From Animal Models

**DOI:** 10.3389/fimmu.2019.02569

**Published:** 2019-11-05

**Authors:** Nonantzin Beristain-Covarrubias, Marisol Perez-Toledo, Mark R. Thomas, Ian R. Henderson, Steve P. Watson, Adam F. Cunningham

**Affiliations:** ^1^Institute of Cardiovascular Sciences, College of Medical and Dental Sciences, University of Birmingham, Birmingham, United Kingdom; ^2^Institute of Immunology and Immunotherapy, College of Medical and Dental Sciences, University of Birmingham, Birmingham, United Kingdom; ^3^Institute for Molecular Bioscience, University of Queensland, Brisbane, QLD, Australia; ^4^Centre of Membrane Proteins and Receptors, Universities of Birmingham and Nottingham, Midlands, United Kingdom

**Keywords:** thrombo-inflammation, pathogens, bacteria, virus, thrombosis, platelets, *Salmonella*

## Abstract

Thrombosis is a common consequence of infection that is associated with poor patient outcome. Nevertheless, the mechanisms by which infection-associated thrombosis is induced, maintained and resolved are poorly understood, as is the contribution thrombosis makes to host control of infection and pathogen spread. The key difference between infection-associated thrombosis and thrombosis in other circumstances is a stronger inflammation-mediated component caused by the presence of the pathogen and its products. This inflammation triggers the activation of platelets, which may accompany damage to the endothelium, resulting in fibrin deposition and thrombus formation. This process is often referred to as thrombo-inflammation. Strikingly, despite its clinical importance and despite thrombi being induced to many different pathogens, it is still unclear whether the mechanisms underlying this process are conserved and how we can best understand this process. This review summarizes thrombosis in a variety of models, including single antigen models such as LPS, and infection models using viruses and bacteria. We provide a specific focus on *Salmonella* Typhimurium infection as a useful model to address all stages of thrombosis during infection. We highlight how this model has helped us identify how thrombosis can appear in different organs at different times and thrombi be detected for weeks after infection in one site, yet largely be resolved within 24 h in another. Furthermore, we discuss the observation that thrombi induced to *Salmonella* Typhimurium are largely devoid of bacteria. Finally, we discuss the value of different therapeutic approaches to target thrombosis, the potential importance of timing in their administration and the necessity to maintain normal hemostasis after treatment. Improvements in our understanding of these processes can be used to better target infection-mediated mechanisms of thrombosis.

## Introduction

### Thrombosis Is a Deadly Complication of Infection in Humans

Systemic or localized infections increase the risk of thrombosis ~2–20 times and are independent risk factors for thromboembolic diseases such as deep vein thrombosis (DVT)/pulmonary embolism (PE) as well as cardiovascular (myocardial infarction) and cerebrovascular events (stroke) (1, 2). The greatest window of risk is when the infection is active, or the weeks shortly afterwards, and in most cases the causative pathogen is not identified (2–5). All types of infection can elevate risk, although some appear to increase risk more than others. Thus, augmented risk of venous thromboembolism is observed in cases of pneumonia (OR, 3.6), symptomatic urinary tract (OR, 2.2), oral (periodontitis and gingivitis–OR, 12), intra-abdominal (OR, 18), and systemic/bloodstream infections (with or without laboratory-confirmed culture–OR, 11–21) (1–3, 6).

There is some discussion about the inter-relationship between infection-induced thrombosis and pre-existing risk factors, such as smoking, lipid levels, and sedentarism, in terms of whether these risk factors directly influence pathogen-driven thrombosis (5). Nevertheless, it is likely that infection promotes the risk of thrombosis in such individuals, although it is important to consider that infection alone is sufficient to drive thrombotic events (7–12). For example, ischemic stroke is associated with acute infections (particularly in the first 3 days after respiratory or urinary tract infection) as are chronic infections such as chronic bronchitis (5). Many common pathogens cause infections that can enhance the risk of thrombotic complications such as stroke. These include–*Helicobacter pylori, Chlamydia pneumoniae, Mycoplasma pneumoniae, Haemophilus influenzae, Strepptococcus pneumoniae, Staphylococcus aureus, Escherichia coli*, Epstein-Bar virus, herpesvirus, and cytomegalovirus (2, 5, 13). Moreover, exacerbations of cardiovascular disease, including acute myocardial infarction and unstable angina, have been observed in bacteraemic infections caused by *Neisseria meningitidis* and *Staphylococcus aureus* (14–16). This association is not limited to adults but is also observed in children in acute conditions such as sepsis, necrotizing enterocolitis, and otitis media; or in chronic pulmonary infections caused by respiratory syncytial virus or *Pseudomonas aeruginosa* (17). Since thrombosis is observed after infection with a diverse range of pathogens, it suggests the ultimate risk of thrombosis after infection is influenced by both host and pathogen-derived factors (15).

The pathological consequences of thrombosis during infection have been extensively studied (18–20). The key factor that underpins the risk of thrombosis is the level of inflammation that is induced by the infection, which drives a pro-coagulant state, with more severe infections promoting greater inflammation and higher risks of thrombotic complications. Sepsis, as the ultimate expression of an un-controlled infection, often occurs without an infective agent being identified. In sepsis there is an excessive systemic inflammatory response syndrome (SIRS), which can lead to multi-organ failure and the death of the patient (21). Sepsis is frequently associated with disseminated intravascular coagulation (DIC), a critical presentation of altered blood coagulation and microthrombus formation in the microvascular bed of different organs (6, 22, 23). The risk of thrombotic complications after infection is not limited to the hospital setting. There is clear evidence that in the community setting, infections increase the risk of venous thromboembolic complications (DVT/PE) (1), with the host and the pathogen both determining the outcome of this relationship (16). In SIRS and DIC, inflammation is mediated by multiple cytokines such as interleukins 1, 6, and 8 (IL-1,−6, and−8), interferons (IFNs) and tumor necrosis factor α (TNFα) (24). Moreover, there is a strong association with damage-associated molecular pattern (DAMPs) molecules like DNA and histones, both as free molecules and within neutrophil extracellular traps (NETs), which are released by activated leucocytes and also promote thrombi formation (25). These combine to promote the pro-coagulant state leading to endothelial damage, platelet activation and aggregation, increases in pro-coagulant proteins such as tissue factor (TF), and reduced activity of anticoagulant mechanisms like fibrinolysis. Compounding this, pathogens themselves are often capable of modulating inflammation and the coagulation system through the production of either pro- or anti-coagulant proteins (26–28). This will be discussed in more detail later in this review.

## Models to Study Thrombosis Induced by Infection

**Figure 1:**
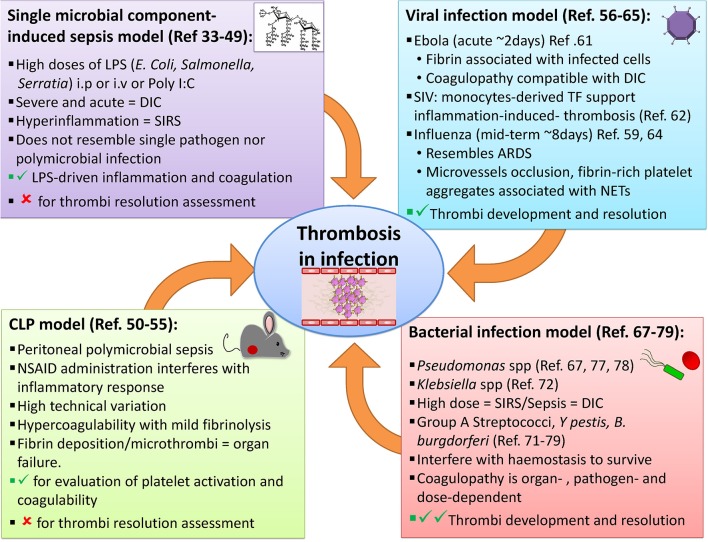
Examples of animal models available to study thrombosis during infection. A range of approaches has been employed to evaluate infection-induced thrombosis. Single microbial component-induced sepsis, or CLP models, mimic severe sepsis in humans, but most are usually only useable for a few days either because the infection is cleared or it is lethal. Some viral and bacterial infection models can induce longer-lasting infections and thrombotic complications may also be useful to study the resolution of thrombosis.

The link between infection and thrombosis has mostly been studied in the context of sepsis. Animal models that study infection-associated coagulopathy typically examine the link between high antigen burdens and the resulting hyper-inflammation, often ignoring other infectious disease-mediated effects on coagulation system. One of the accompanying advances that has helped in interpreting the events revealed by these models, has been the improvements in imaging thrombosis and infection. In particular, the advent of more advanced microscopy techniques, such as intravital microscopy, has contributed to a better understanding of how the events associated with infection-induced thrombosis occur in real-time. Through these techniques, pathogen-host cell interactions can be tracked *in vivo* in multiple tissues (29–31). These transformative approaches have underpinned a new understanding on how multiple cell-types, such as neutrophils and platelets, interact to generate thrombi, and on occasion, bind to pathogens. Below, we summarize and discuss different models of infection and thrombosis (Figure 1), with a particular focus on the potential of these models to study not only the triggering of thrombosis but also its development and resolution.

### Single Microbial Component-Induced Sepsis Models

Animal models of sepsis induced using a single product derived from pathogens are typically based on the recognition of pathogen-associated molecular patterns (PAMPs) by immune cells during infection. These models attempt to resemble the human septic state by injecting a single pathogen-derived molecule, either intraperitoneally (i.p.) or intravenously (i.v.). The endotoxemia model induces systemic inflammation through administration of lipopolysaccharide (LPS), purified from different gram-negative bacteria (e.g., *E. coli, Salmonella*, or *Serratia*), given in doses much higher (1–10 mg/kg) than the amount potentially found in bacteria during a more realistic septic state (32). LPS is a major component of the outer cell wall of gram-negative bacteria and is recognized by host cells through multiple pathways including toll-like receptor 4 (TLR-4) (33). The fact that LPS is not a true toxin, but a cell wall component involved in maintaining structural stability, highlights how the host-derived responses to bacterial components, rather than their intrinsic toxicity, is important in driving the pro-thrombotic state. Binding of LPS by host cells such as monocytes/macrophages, platelets and endothelial cells results in a pro-coagulant state through directly activating pro-inflammatory responses concurrently with coagulation factors like monocyte-derived tissue factor (mTF) within the vasculature (34). Among the inflammatory mediators released in this sepsis model are IL-1, IL-6, TNFα, and IFNγ which can induce or potentiate leukocytes and endothelial cells to upregulate pro-coagulant molecules such as TF, FVII/FVIIa, and thrombin driving thrombi generation (35). Moreover, these pro-inflammatory molecules also reduce the levels of endogenous anti-coagulant proteins, like tissue factor pathway inhibitor (TFPI), antithrombin (AT), and activated protein C (APC) (36). Together this increases the thrombotic risk of consumption pathology like DIC (37).

The LPS model has demonstrated the association between inflammation, platelet activation, and coagulation (38) and the potential of targeting the inflammatory cascade to reduce DIC severity and mortality (34, 36). However, few studies have examined if the resultant thrombi are efficient traps for LPS. A comparative study done with human and mouse blood as well as arthropods hemolymph, showed that the blood clot binds LPS *in vitro*, during or shortly after clotting (39). This study also demonstrated binding of LPS to platelets and fibrin within the clot *in vivo*, using a mouse model of laser-induced thrombosis in the cremaster muscle and an infusion of labeled-LPS (39). Thus, thrombi in some circumstances seem to be able to trap LPS and this has helped evolve the concept that thrombi can also trap bacteria (40).

Modest doses of alpha toxin (1 μg/mouse i.v.) have been used to model staphylococcal sepsis (41, 42). Furthermore, in a later phase (4–8 h after infection), alpha toxin secreted during *S. aureus* bacteremia, is directly responsible for platelet aggregation in the microvessels. These thrombotic events in the microvasculature lead to organ dysfunction in liver and kidney correlating with disease severity. The approach used in this study (43), although acute, allows investigation of the early events during septic coagulopathy and the direct effect of pathogen products on hemostasis, and reveals the possibility of targeting the alpha toxin rather than a cell receptor therapeutically.

Polyinosinic:polycytidylic acid (Poly I:C) is a synthetic double-stranded RNA that interacts with TLR-3 and is used experimentally to mimic aspects of viral infections (44, 45). TLR-3 expression on platelets and megakaryocytes has been reported and its ligation on platelets can potentiate platelet aggregation by other classical platelet agonists (46). The use of Poly I:C for studying thrombosis is not as widespread as for other models, but injection with Poly I:C has been described to induce TLR-3-dependent arterial thrombosis, but a more minimal thrombosis in the venules (47). As there is limited data for the use of Poly I:C in this context, more work is needed to better understand the strengths and limitations of its use to recapitulate what is observed after viral infection.

One limitation in these single-agent models is that they typically induce a highly acute systemic organ failure through the inflammation-induced deposition of fibrin in multiple organs (48). The use of single components also underplays the complexity of pathogens, which typically can induce multiple innate and adaptive pathways concurrently. It also does not take into consideration other potentially pro-thrombotic antigens that may be present in the pathogen, nor the active role that live bacteria themselves may play during sepsis (28). Moreover, during sepsis, concurrent with fibrin deposition, other systemic events are ongoing, including lowering of the blood pressure, increases in the heart and respiratory rate, arterial hypoxemia, as well as derangement of white blood cell and platelets counts. In combination, these can lead to organ dysfunction and in the most severe cases progress to septic shock and death (49). Moreover, despite its usefulness, the pathways identified using the LPS model have not translated into clinical benefit to date. Thus, single microbial component-induced sepsis models have intrinsic limitations for studying specific complications of infection such as thrombosis development or its resolution.

### The Cecal Ligation and Puncture Model

The cecal ligation and puncture (CLP) model evaluates polymicrobial sepsis through the introduction of fecal contaminants into the peritoneal cavity after perforation of the cecum (32). This model resembles the peritonitis observed during a number of conditions such as appendicitis, diverticulitis, bowel perforation, and trauma. After puncture, a complex bacterial community translocates from the gut into the host. Included within this population are well-studied organisms such as different *E. coli* strains and other Gram-negative bacteria. In addition, there are many other bacterial genera and species present, many of which are difficult to culture. This means that although the model recreates what is observed in some clinical conditions, it can be difficult to fully appreciate the diversity of bacterial species and the total numbers of bacteria present. Furthermore, the composition of the gut microbiome should be taken into consideration when using this model as it may influence outcome. For instance, it has been reported that C57BL/6 mice from two different suppliers show differences in their survival following CLP. The immunophenotype and the level of inflammation in these animals differed after CLP. The supplier-associated effects were no longer detectable if the animals were co-housed. Thus, the microbiome can influence survival in this model (50).

After CLP, animals develop hypercoagulability with mild fibrinolysis. Only a few reports have studied thrombi formation in this model and these typically show fibrin deposition or microthrombi formation in organs such as kidney and liver (51, 52), highlighting the impact of coagulopathy during severe infections. Indeed, the development of thrombi rich in platelets in the microvasculature of kidney, lung, and liver has been observed 48 h after CLP, correlating with organ dysfunction due to ischemic complications such as kidney failure and respiratory distress syndrome (53). Moreover, sepsis-induced thrombocytopenia is attributed to the sequestration of platelets either within thrombi or complexed with leukocytes (54). Thus, the CLP model has been used to evaluate platelet activation and coagulability during peritoneal sepsis. In the CLP model, mice typically do not recover and combined with its relative acute nature (a few days) and the variability of the bacterial burden resulting from puncture, this means that it has limited value to study thrombus resolution despite its clinical relevance (52, 55).

### Viral Infection Models

Many viral infections target hemostasis and coagulation, introducing either hemorrhagic or thrombotic complications (56). Indeed, in humans, seasonal flu vaccination can moderate the risk of some cardiovascular events (57). Among the viruses associated with thrombotic complications in humans are exanthematous viruses such as varicella, variola, measles, and vaccinia; arboviruses like dengue virus; ebola virus; and also influenza virus, hepatitis, HIV, and cytomegalovirus (58). A potential limitation here is that many viruses demonstrate exquisite host specificity meaning that equivalent viruses are not always available for study in animals.

There is strong evidence showing abnormal hemostasis linked to inflammation during viral infections in humans (8, 9, 59, 60). In animals, some viral infections like Ebola and influenza have been used to study thrombosis. Macaques inoculated intramuscularly with 1,000 plaque forming units (pfu) of Ebola virions, not unsurprisingly develop coagulation abnormalities compatible with DIC from day 2 after infection (61). In this study, Ebola virus (EBOV) infection induced the expression of TF in mononuclear phagocytes, triggering activation of the coagulation system (61). Moreover, this work showed histological evidence of fibrin deposits in tissues (spleen, liver, and kidney) from EBOV-infected macaques. The fibrin deposits were in association with activated macrophages and in close contact with infected cells and viral proteins, thus highlighting the interplay between inflammatory cells and coagulopathy. Monocyte-derived TF contributing to inflammation-induced thrombosis was also demonstrated in a Simian virus immunodeficiency model linking HIV infection with cardiovascular complications (62).

In a model of acute respiratory distress syndrome (ARDS), mice infected intranasally with a lethal dose of influenza virus were shown to have increased platelet aggregation, pulmonary microvascular thrombosis, endothelial damage and hyper-inflammatory cytokine responses (59, 63, 64). Furthermore, occlusion of microvessels with fibrin-rich platelet aggregates and the presence of extracellular histones within those aggregates was shown, reflecting what has been described in endotoxemic and CLP models of sepsis (25, 65). This model linked the prothrombotic effects of inflammation through NETs and potentially through the secretion of platelet-derived soluble CD40 ligand (sCD40L), which can induce platelet aggregation (66). Interestingly, over 95% of sCD40L in circulation comes from platelets, which might contribute to thrombosis during infection due to its capacity to promote histone-platelet interaction (65). Mice lethally infected with influenza virus survive no more than 8 days post infection (d.p.i.) and thrombosis was observed from day 3 p.i. (65); making this model more suitable for the study of thrombi development under highly inflammatory conditions and its potential implications in the physiopathology of complications such as ARDS. Thus, viral infection models such as influenza can be used to have a better understanding of the mechanisms of thrombosis promoted by these pathogens.

### Bacterial Infection Models

Multiple models use live bacteria to induce SIRS and the associated thrombo-inflammatory consequences, mimicking what happens during conditions such as sepsis (32). However, few animal models involve bacterial infections to evaluate how a long-term established infection, and the associated inflammation, may impact on the coagulation state of the host. Most *in vivo* models inoculate intravenously with relatively high doses of bacteria (from 1 × 10^7^ to 2 × 10^9^ CFU (Colony Forming Units/mL, depending on the model) (67–69), which can lead to a massive induction of complement-dependent bacteria lysis and antigen release. Live and killed bacteria can activate a wide range of innate receptors known as pattern recognition receptors (PRR), such as Toll-like receptors (TLR), NOD-like receptors (NLR), and others (33). These models have informed on how pathogens can actively modulate not only the immune response, in order to escape control, but also the coagulation cascade to favor bacterial invasion (70). Examples of pathogens used in such studies include *S. aureus, Pseudomonas aeruginosa*, group A Streptococcus, *Klebsiella pneumoniae, Yersinia* spp, *Bacillus anthracis, B. cereus*, and *Salmonella* Typhimurium, among others (67, 68, 71–75).

The speed of the host response is striking. Within the first 30 min of infection with an outbreak strain of *S. aureus* or another Gram-positive organism, *Bacillus cereus*, platelet aggregates are bound to Kupffer cells in the liver (43, 75). These aggregates can help enhance bacterial capture and limit bacterial dissemination, demonstrating the potential value of platelet interactions with host cells in host defense. Several studies using a *P. aeruginosa*-induced model of ARDS have evaluated the pro-coagulant imbalance during acute lung injury that has been associated with decreased fibrinolytic activity (67). These models highlight the capacity of early fibrin formation to limit damage, but its potential to harm if it persists (76). This led to the proposal that modulation of hemostasis, either by administration of recombinant human antithrombin (rhAT) or recombinant human activated protein C (rhAPC), may restore the fibrinolytic activity, then limiting the amount of inflammation (67, 76). Administration of rhAPC treatment or fibrin-derived peptides (Bβ 15-42) can exert a protective effect during acute lung injury (ALI), without modifying the inflammatory response to *P. aeruginosa* nor the bacterial clearance (77, 78). Moreover, in a different model of pneumonia induced by *K. pneumoniae*, overexpression of human tissue-type plasminogen activator (t-PA) was associated with a higher level of fibrinolysis in the lungs and decreased thrombus formation in the liver and subsequent improvements in survival of the host (68). These studies show the importance of fibrinolysis during infection and the roles coagulation/fibrinolytic factors can play in severe infections depending on the location of the infection and the pathogen.

Multiple human pathogens express coagulation-related proteins, such as Streptokinase (SK) produced by group A streptococci (GAS) and Staphylokinase from *Staphylococcus aureus* (71), which activate plasminogen resulting in increased fibrinolysis. These proteins are similar to the Pla protein expressed by *Y. pestis* (26), which can activate plasminogen as well as mediate bacterial adhesion to extracellular matrices aiding spread throughout host tissues (28). Other microorganisms, such as *Borrelia burgdorferi*, use the host plasminogen receptor to enhance migration though tissues (27, 28). Thus, different animal models can help understand how pathogens interact with the coagulation system and how this impacts on the pathogenicity during infection. GAS are human pathogens that cause a variety of infections, from mild pharyngeal and skin infections to necrotizing fasciitis and toxic shock-like syndrome (71, 79). GAS are human-specific pathogens, with limited animal models available to use for their study, although humanized transgenic mice have been used successfully to study host-pathogen interactions *in vivo* (71). These organisms can interfere with hemostasis to promote their survival and dissemination, partly through sequestering host plasminogen. This is then converted into plasmin, which results in degradation of the fibrin network that helps contain the bacteria, enabling escape from the clot and dissemination to other sites within the host (71, 79–82). Fibrin can have an important role in limiting pathogen invasion at sites of injury. This was demonstrated, using *in vivo* microscopy, in a wound infection model using *P. aeruginosa* in mice. This showed that fibrin mesh acts as a physical barrier to prevent bacteria from penetrating the wound, while a full clot forms a permanent seal (83). Thus, the fibrin wall induced rapidly after trauma enhances resistance to infection.

As mentioned previously, most animal models of sepsis use Gram-negative bacteria or their endotoxins to evaluate coagulopathy. A limitation of these models is that they provoke an acute endotoxemia, rather than a sustained infection. Notwithstanding this temporal constraint, these models have been used to evaluate the potential for thrombosis to aid host defense. After systemic infection of neutrophil serine protease-deficient mice with relatively high doses of *E. coli* (3.2 × 10^8^ CFU), fibrin deposition and restriction of bacteria within the sinusoid vasculature is reduced and there is an increase in bacteria within tissues (31). Since neutrophil proteases are essential to induce NETs, the authors concluded that the reduction in fibrin deposition (microthrombi) is directly responsible for bacterial spreading into the liver. Whilst indicative of a role of NETs in restricting bacterial dissemination, other roles for serine proteases may also contribute to these effects.

Studies have also examined responses using far lower doses of bacteria in acute or chronic infection models (72, 73, 84). An example of this is a mouse model of *Bacillus anthracis* infection (5 × 10^6^ spores per mouse), which was used to study thrombus formation and composition in the liver in anthrax. The authors described thrombi in the liver microvasculature and this was associated with lethality. Most thrombi were composed of platelets, leucocytes, von Willebrand factor (VWF), Syndecan-1 and fibronectin (73). They attribute the bacteria-induced endothelial injury to the clot formation. Moreover, the presence of bacteria aggregated with platelets and leukocytes supports the previously described role of inflammation in infectious coagulopathy and bacterial trapping (85). Proteins such as VWF, collagen and fibronectin contribute to the hemostatic response and to endothelial damage. Furthermore, a potentially protective role for different pro-coagulant mediators such as fibrin, TF, plasminogen activator inhibitor-1 (PAI-1) and TAFI have been described during *Y. enterocolitica* infection (5 × 10^4^ CFU) in the mouse. Although the mechanism needs to be elucidated, this study suggests that activation of coagulation during infection induces innate responses that further activate multiple coagulation pathways. Thus, activation of the coagulation cascade might be required for an efficient host immune response against some Gram-negative bacteria (72). Collectively, these studies demonstrate the close relationship between bacterial pathogens, hemostasis and coagulation and offer tantalizing evidence that thrombi can help restrict bacterial dissemination.

Based on these studies, it is important to recognize that not all the infections are the same and that the role of coagulation during bacterial infections can vary in a pathogen- and dose-dependent manner. Understanding how pathogens, coagulation and inflammation interact will allow exploration of alternative therapeutic targets that offer better outcomes than the current ones.

## *Salmonella* Infections of Mice: a Model to Evaluate the Induction and Resolution of Infection-Mediated Thrombosis

### *Salmonella* spp. and the Infection They Cause

*Salmonella enterica* (*S. enterica*) are gram-negative, facultative intracellular microorganisms that cause hundreds of thousands of deaths each year, with risk of death increasing markedly when bacteria invade systemically. There are more than 2,500 serovars of *S. enterica*, and these can infect a wide range of species, including humans, other mammals, and livestock. Many human infections are zoonotic in origin, such as those caused by the serovars *S*. Typhimurium (STm) and *S*. Enteritidis (SEn), though some serovars, such as *S*. Typhi, or *S*. Paratyphi A-C, the causative agents of enteric fever, are human-restricted. Non-typhoidal *Salmonellae* (NTS), such as STm and SEn, typically cause acute, self-limiting gastrointestinal infections. However, malnutrition, immunodeficiencies and co-infection with malaria or HIV, increase the risk of acquiring invasive non-typhoidal salmonellosis (iNTS), which is a systemic infection with high mortality rates of up to 25% (86).

A relationship between typhoid and thrombosis has been known for over a century (87, 88). There are also several reports of cases where patients infected with *Salmonella* present with thrombosis as a complication (89–93). Thrombosis caused by *Salmonella* infections has also been reported in animals, such as calves (94), rats (95), and hamsters (96). Collectively, such studies demonstrate that *Salmonella* infections are able to induce thrombosis in a broad range of animal hosts.

### Mouse Models of *Salmonella* Infection

STm infection of mice is a promising model for studying thrombosis development and resolution during infection. When injected into mice, STm induces an infection that resembles invasive disease (aspects of enteric fever and iNTS). STm can be given i.v., i.p., or orally, and mice from different genetic backgrounds can be used. The mouse model of STm infection has one major advantage over many other bacterial infection models—mice can be infected with sufficient numbers of bacteria to induce quite marked and prolonged clinical signs, but the mouse will ultimately resolve the infection over a period of weeks.

In mice, susceptibility to *Salmonella* infection is associated with the gene Solute Carrier Family 11 Member 1 (*Slc11a1*), which encodes for the natural resistance-associated macrophage protein 1 (hereafter referred to as Nramp1). Nramp1 transports divalent cations out of the phagosomes, which interferes with the function of bacterial enzymes required for the survival of many intracellular pathogens, including *Salmonella* (97–99). A single point-mutation in Nramp1 markedly reduces the LD_50_ (Lethal Dose, 50%) of a mouse to virulent STm >1,000-fold. Therefore, it is important to consider the bacterial strain, the mouse strain and the route of infection in this model. Mouse strains with a missense mutation in Nramp1 include the commonly used C57BL/6J and BALB/c strains, the background for most genetically-altered mice. Infection of these strains either i.p. or i.v. with <10 CFU of a virulent STm strain (e.g., the commonly used laboratory strain SL1344) leads to an acute and systemic infection with bacteremia, where bacteria reach ≥10^6^ CFU/mL blood, and is fatal in <7 days. In contrast, resistant strains with functional Nramp1, such as CD1 and Sv129S6 strains, are more resistant to infection with this strain, are able to clear or develop long-term chronic colonization (99). Susceptible mouse strains can be infected orally with higher numbers of virulent bacteria, but if the infection goes systemic then a similar outcome to i.v. infection will be observed.

Few genetically-altered mice are on resistant backgrounds, which can limit the possibility to perform longer-term mechanistic studies without undertaking expensive and time consuming backcrossing of mice. Such limitations can partly be overcome by infecting susceptible mice i.p. or i.v. with attenuated STm [e.g., aroA-deficient SL3261 (100)]. Using such bacteria, it is possible to inoculate with higher bacterial doses (typically 10^5^-10^6^ bacteria) per mouse, with the infection resolving over a period of months [e.g., (101, 102)]. After infecting susceptible mice with this dose either i.p. or i.v., bacteria can be retrieved from the bone marrow, brain, kidneys and lungs, but the organs with the highest bacterial burdens are the spleen and liver (74). In both organs, bacterial numbers peak at the end of the first week and fall gradually from the third week post-infection, to ultimately be cleared by around 8 weeks (101), and co-infection can influence the rate of clearance (103).

The inflammatory cell response that follows STm infection is dominated by monocytes and neutrophils. At 24 h after i.p. infection of C57BL6/J mice with attenuated STm, we have found that in the spleen, there is an increase of in the total number of Ly6C^+^ monocytes (104). Additionally, Tam et al. have shown that, after intragastric administration of the bacteria, at 7 days post-infection, there was an increase in the total number of CD11b^+^Ly6C^hi^ and Ly6C^int^ cells in both spleen and liver (105). Infection with STm also induces inflammation in the liver. However, this is organized in granuloma-like structures, often referred to as inflammatory foci. These foci consist mainly of F4/80^+^ cells and have been shown to be sites of bacterial containment (106). Neutrophils have been detected in the peritoneum of infected mice as early as 12 h post-injection (107) and they have shown to accumulate in sites of infection, such as spleen and liver (105). Together these studies suggest that there are contributions from tissue-resident and migratory innate immune cells to the inflammatory response after STm infection in different tissues. This can help explain why there are different inflammatory responses at different sites at different times after infection that in turn may impact on the response to the bacteria.

### Thrombosis and *Salmonella* Infection in Mice

Thrombosis induced after STm infection has been reported in both resistant and susceptible strains of mice after infection with virulent and attenuated strains of bacteria, respectively. After infecting Sv129S6 mice orally with a virulent strain of STm (SL1344), Brown *et al* described the development of hepatic microthrombi after the first week of infection, followed by a more extensive thrombosis in the spleen and liver at 3 weeks post-infection (84). Furthermore, venous thrombosis in the livers of mice can be observed months after infection, although levels were not quantified, meaning it is difficult to judge their prevalence long-term (108). Moreover, in a mouse that died from infection, there was a marked thrombosis in the spleen and liver, suggesting that this correlates with disease severity. Vaccination can reduce the level of thrombosis induced (109). Furthermore, a model to study *Salmonella*-associated meningitis demonstrated that this infection can induce thrombosis in the brain (110).

### Thrombosis After STm Infection Is Driven by Inflammation

Our own interest in studying thrombosis after STm infection came from our observations on the effects of STm infection on immune cell populations and host immune homeostasis. Using a model of infection of susceptible mice with attenuated STm we had identified how infection altered the functioning of sites such as the bone marrow and thymus (111, 112). When we examined the response in the liver (74), it was dramatically different to control mice. By 7 days after infection, there was a substantial recruitment of monocytes into inflammatory foci accompanied by the parallel presence of an extensive thrombosis. Thrombi were overwhelmingly restricted to the hepatic portal vein, were white in appearance and contained mainly platelets, fibrin, and immune cells. They were typically observed at sites of endothelial perturbation. Additionally, the levels of thrombosis and inflammatory foci in the liver are maintained at their peak levels between 1 and 3 weeks post-infection despite bacterial numbers in the organ falling by 90–99% during this time and bacteremia not being detected. The link between inflammation and thrombosis was confirmed by studies that showed that after infection of mice deficient in TLR4 or IFNγ, there was a near absence of both thrombosis and inflammatory foci. This supports the concept that thrombosis is triggered by the infection but driven by the inflammatory response (74).

The association between disturbed endothelial integrity and thrombus development suggests that thrombi form after contact with sub-endothelial cells or other factors that are absent in healthy tissues. In the liver, STm infection induces the upregulation of podoplanin in inflammatory foci and podoplanin can bind C-type lectin-like receptor 2 (CLEC-2) that is expressed on platelets and can mediate platelet activation (113). Transgenic mice lacking CLEC-2 on platelets and megakaryocytes (PF4.Cre-Clec-Ib^fl/fl^) had much lower levels of thrombosis, indicating that CLEC-2-podoplanin ligation drove platelet activation and subsequently thrombosis (74). Loss of CLEC-2 expression in these cells did not significantly alter bacterial clearance or the capacity to drive inflammatory foci development. Furthermore, treatment of mice with clodronate liposomes, which reduces numbers of macrophage/monocytic cells, abrogated the development of thrombosis in infected mice. Therefore, using the murine model of STm infection, a novel mechanism of infection-mediated thrombosis was identified (74).

### Infection Can Develop in Spleen and Liver With Discordant Kinetics

Our original studies had focused on the liver since at day 7 post-infection thrombosis was extensive in this organ, with thrombi located in the portal vein. Parallel assessment of the spleen, which has a similar peak bacterial burden and rate of clearance, had not identified such an extensive thrombosis and so it was initially considered that the liver was the major site where this developed. Thus, it was unexpected when we observed that in the same model of infection a widespread thrombosis was detected in the spleen 24 h after infection, with thrombi localized to the splenic vein (104). In both organs, thrombi are not detected to any significant degree in the arterial system. It should be noted that to observe thrombi, particularly in the bigger vessels, then it is important to section deeply into the tissues, and since the spleen can increase in size >10-fold post-infection, this is can be technically challenging. In the spleen, it is optimal to section longitudinally to the hilum to ensure better representation of the splenic vein on a section. Thrombosis in the spleen was transient since thrombus numbers and size had fallen dramatically by 48 h post-infection and remained low thereafter. Therefore, thrombosis can occur in different organs at different times post-infection despite both those organs containing similar bacterial burdens. Another lesson we can learn from this is that the resolution of thrombosis, at least in the spleen, does not require a fall in bacterial numbers inside an organ, since in this model, bacterial numbers are stable or rising in the first days after infection (104).

**Figure 2:**
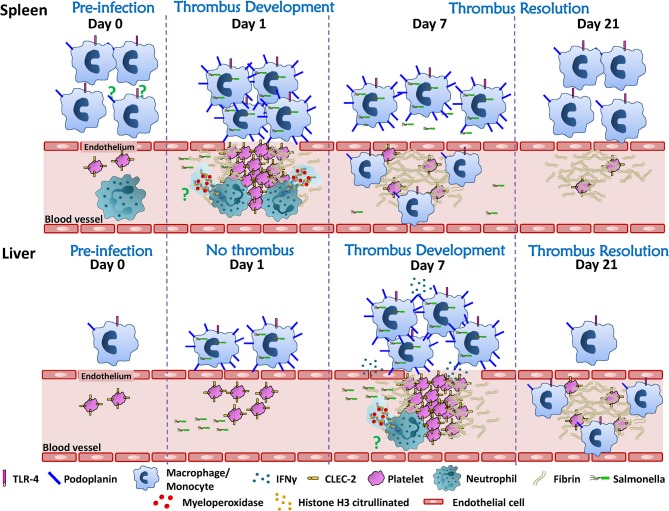
Thrombosis develops asynchronously in the spleen and liver after STm infection. Resident immune cells (monocytes/macrophages/neutrophils) are more abundant in the spleen compared to the liver. Although the mechanism is still under study, splenic phagocytic cells and neutrophils participate in the induction of white, platelet-rich, thrombi in the spleen 24 h after infection. Thrombi in the spleen resolve rapidly thereafter so that from day 7 onwards post-infection only “ghosts” of fibrin are observed. In the liver, thrombi are not detected until 7 days after infection, despite the liver and spleen having similarly high bacteria burdens in the first week post-infection. Thrombosis in the liver requires inflammatory cell recruitment and activation through TLR4, which promotes IFNγ production, the accumulation of monocytic cells and their upregulation of podoplanin. Inflammation is associated with perturbed endothelial integrity/endothelial damage making it possible for the interaction of podoplanin expressed on monocytic cells and CLEC-2 on platelets. Thrombosis in the liver persists for weeks and the resolution of thrombosis is observed from day 21 after infection. The mechanism(s) that underlie the resolution of thrombosis in these two different organs is still unknown. A strength of the STm infection model is that it offers an alternative tool to understand this process.

The mechanism of thrombosis in the spleen is less explored. Phagocytic cells such as monocytes/macrophages play an important role, since depletion of these cells by clodronate treatment prior to infection, resulted in a significant reduction in numbers of thrombi in the spleen (104). The exact contribution of different monocytic-lineage cells to this process is currently unknown, but with subpopulations of such cells occupying distinct niches in the spleen such as the red pulp or marginal zone, it is possible that each subpopulation make distinct contributions to this process. However, the fact that thrombosis in the spleen follows such a discordant kinetics compared to the liver, both in induction and resolution, suggests that there may be additional site-specific mechanisms involved, as summarized in Figure 2. An additional contribution to the risk of thrombosis in different sites may be the level of local endothelial activation, but this has not been explored in great depth. Further experiments exploring the cellular requirements and the cytokines that may contribute to the establishment of thrombosis in the spleen are currently undergoing. Details revealed using longer-term infection models such as STm could help us understand the relationship between thrombosis and infection within a specific organ, or alternatively, the findings may only be specific to STm infections. This could ultimately help us identify how to predict where thrombosis will develop for a specific infection, if the agent is unknown or if no agent is identified then it might still be possible to predict sites of thrombosis if the same mechanisms are conserved in other systems. A second implication is that if there are multiple, site-specific mechanisms active to the same pathogen, and this holds true for multiple infections, then it may have significant implications for trying to control thrombosis. This is because it would imply that a single, one-size-fits-all approach to treatment is not likely to be useful in all cases, and may help explain the limited success observed in controlling infection-associated thrombosis to date. Thus, it is likely to be important to study the kinetics of thrombosis in multiple organs to understand all the mechanisms for triggering thrombosis and its resolution.

An interesting contrast between the *Salmonella* model and that of other bacterial infection models is the timing when thrombosis is detected. As described above, in most bacterial infection models using Gram-positive or negative bacteria thrombosis is detected in the first minutes or hours after infection, rather than much later for *Salmonella*. This indicates the importance in identifying the most appropriate model to use to address the researchers scientific question. Nevertheless, it is possible that infection with STm can induce thrombi this rapidly, and such effects may be more readily identifiable with infection at higher doses of bacteria than those typically used, but we have not been able to identify a report where this has been done. Microthrombi in the liver have been reported, presumably arterial, in mice one week after infection, but their location was not specified (84). Potential reasons for the striking differences in timing are worth considering. The later onset may simply reflect the non-lethality of the *Salmonella* model when used as typically described in the literature, meaning productive infections are active for longer. The numbers of *Salmonella* given are relatively low, typically <1%, compared to the number of *E. coli* bacteria typically given, and so it may take longer for thrombosis to be induced. If other bacterial infections could persist to similar levels, and for as long as *Salmonella*, then perhaps a similar pattern of thrombosis would be observed. Indeed, there are many examples of cases of thrombosis that occur in humans and primates when the infection is known to have been established for days. For instance, in a primate model of pneumonic plague, where death occurred between 3 and 7 days post-challenge, then widespread thrombosis was detected in the kidneys, similar to what has been described elsewhere for natural *Salmonella* infections in hamsters (96, 114). The temporal window for detecting thrombi detailed in these examples and for STm in the mouse show some overlap, indicating that thrombosis can occur at similar times in other model systems and so may be reasonably common. Nevertheless, there needs to be caution in taking these analogies further, which ultimately highlights the need for further studies in animal models and in humans. Although, the observations in mice after STm infection may be analogous to what is observed elsewhere after long-term infection, it still remains possible that the murine model of salmonellosis is unique, or atypical. Furthermore, the nature of the thrombi themselves needs to be considered. Typically, thrombi that form in the venous vasculature commonly are rich in erythrocytes, and often termed “red thrombi.” In contrast, thrombi in the arterial circulation mostly contain platelets and are referred to as “white thrombi.” After STm infection in mice, the thrombi are white, yet located in the venous circulation. What this actually signifies is unclear, and the composition of thrombi post-infection is not particularly well documented even in reviews of autopsies on septic patients (115), despite its importance for directing appropriate treatment (116). All of these points indicate the need for further work to understand the relationship between inflammatory cells in the parenchyma, platelets, and the vessels where thrombi form.

### Thrombi in Mice After STm Infection Contain Few Bacteria

The development of techniques to identify bacteria in infected tissues has enabled us to address directly what level of bacterial load is found in individual thrombi. Based on previous studies (e.g., see above), we had anticipated that there would be significant burdens within each thrombus. Thus, it was unexpected to find that most thrombi contain few or no bacteria. This was the case if splenic or liver thrombi were examined or whether this was early or later after infection (104). This is unlikely to reflect bacteria being rapidly killed and cleared as the methodology used detects antigen and not live bacteria, and there is no difference in the level of detection of bacterial antigen in the splenic thrombi, where thrombosis occurs in a highly synchronized manner, or in the liver, where thrombosis is more protracted. In contrast to this lack of detection of bacteria in thrombi, bacteria were widespread throughout the tissues and always easy to identify. This would suggest that thrombi induced after infection are not always able to act as efficient bacterial traps. Indeed, it could be possible that in many instances trapping bacteria is not a key consequence of the induction of thrombi after infection.

## Current and Potential Therapeutic Targets for Infection-Induced Thrombosis

**Figure 3:**
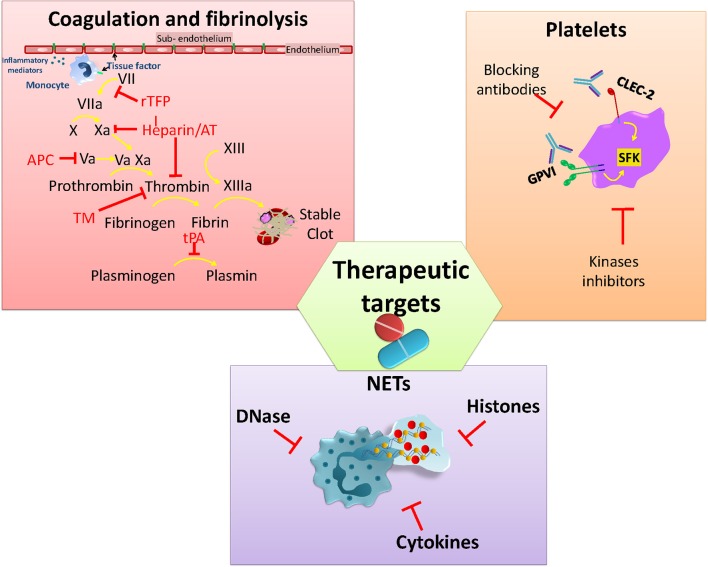
Current and potential molecules or pathways to target infection-associated thrombosis. Anticoagulants interfere with the activity of different clotting factors in the coagulation cascade that aims to rebalance hemostasis. Platelet GPVI and CLEC-2 are activated via SFK kinases and play a role in infection-mediated platelet activation. Neutrophil activation and the release of NETs together with associated factors (DNA/Histones) are also under study for developing strategies to limit their pro-thrombotic effects.

The potential harm caused by thrombosis during infection, and its long-term consequences, has led to it being a target for therapies that either prevent or limit infection-associated thrombosis. Although patients with sepsis will generally receive low molecular weight heparin to prevent DVT/PE there is no current antithrombotic treatment schemes used specifically for patients with infection. Despite the significant attention this area has received, no optimal approach has yet been identified. In this last section, we briefly discuss antithrombotic approaches so far tested, before introducing new potential targets for treating infection-driven thrombosis (Figure 3). The ideal treatment profile would include the capacity to target thrombosis, but not increase the risk of bleeding or worsening infection.

### Coagulation and Fibrinolysis

Anticoagulants have been used during sepsis-induced coagulopathy due to evidence of perturbed levels of pro- and anticoagulant factors in sepsis. The aim is to restore plasma levels of the coagulation factors consumed during sepsis (117). Among the factors so far tested are antithrombin (AT) which blocks thrombin along with other clotting factors (Xa, IXa, VIIa, XIa, and XIIa), thus interfering with fibrin conversion (118). Recombinant TFPI (rTFPI) has been used to block the activation of factor X by Factor VIIa-TF complex, preventing spontaneous activation of coagulation (118). Thrombomodulin (TM) recombinant protein has been used in sepsis to reduce coagulation through binding to thrombin and enhancing protein C activation (119). Activated protein C (APC) targets inflammation and coagulation pathways, inhibiting factors Va and VIIIa to reduce thrombin generation and degrade histones from NETs (118). Recombinant tissue plasminogen activator (tPA) has been used for facilitating thrombus clearance, increasing fibrinolysis during venous thromboembolism, but with limited use in infection-induced coagulopathies (120–123). However, despite these approaches being based on high quality science, all of the anticoagulant recombinant proteins failed to demonstrate a significant benefit during clinical trials and also increased the bleeding risk (124–128). Currently, the use of heparin is attracting significant attention as its activity is associated with relatively low risk of bleeding (129). Heparin targets coagulation through the binding and activation of AT to inhibit thrombin and other clotting factors (IIa and Xa) (118, 130). Moreover, heparin can also limit inflammation by disrupting NETs through interactions with histones (131). Heparin use is widely used for the prevention and treatment of thromboembolic events in many circumstances (130). No published studies have evaluated the use of heparin to limit thromboembolic complications associated with infection.

### Inhibiting Platelet Activity

Platelet activation plays a crucial role not only in infection-driven thrombosis but also during the immune response against pathogens (132). Both infection and inflammation can directly or indirectly activate platelets through different receptors, triggering aggregation and thrombi formation within the vasculature (132, 133). In addition to this, there are some receptors that have attracted the attention of researchers due to their participation in thrombo-inflammation events. Glycoprotein VI (GPVI) and CLEC-2 are two platelets receptors that play roles during infection, either directly in the pathogen response or in infection-driven thrombosis (52, 74, 134, 135). GPVI mediates platelet activation by collagen but it also binds to a number of additional endogenous and exogenous ligands including laminin, fibronectin, adiponectin, toxins, and polysulfated sugars (136). Activation of GPVI has been associated with a protective role during *K. pneumoniae*-induced pneumosepsis through promoting platelet-leukocyte aggregates and enhancing bacteria phagocytosis *in vitro* (134). CLEC-2 interacts with podoplanin, which is expressed by many cell types including podocytes, lymphatic and lung epithelial cells, and inflammatory macrophages (137). As mentioned previously, CLEC-2/podoplanin interactions play an important role in thrombosis in the liver after STm infection of mice (74). Moreover, either genetically deleting or blocking the CLEC-2/podoplanin axis with monoclonal antibodies has shown a reduction in thrombus formation in a mouse model of DVT (138). Similar outcomes are reported in an ARDS mouse model (139) where treating mice with an anti-podoplanin antibody improves the response by altering the cytokine production and cell recruitment. In contrast, CLEC-2-deficiency worsens outcome in the CLP model (52) associated with increased levels of pro-inflammatory cytokines, reduced macrophage recruitment to the peritoneum and higher bacterial numbers at this site. Thus, CLEC-2 and GPVI are potential therapeutic targets for infection-associated thrombosis that would have a limited impact on hemostasis.

Alongside blocking extracellular receptors to inhibit platelet activation, targeting signaling pathways involved in thrombo-inflammation has been proposed as an alternative approach to tackle inflammation-induced coagulation (140). Signaling through tyrosine kinases such as the Tec kinase Bruton-tyrosine kinase (Btk), members of the Src family of kinases (SFK) (Syk, Fyn, Lyn), and spleen activated kinase (Syk) is essential for regulation of hemostatic and inflammatory processes (141). All three families are highly expressed by platelets and leukocytes and have essential role in activation, adhesion, recruitment and effector responses. On platelets, GPVI and CLEC-2 receptors are activated via (hemi)ITAM phosphorylation through Src, Syk, and Tec kinases, making this pathway an attractive target for blocking platelet activation (141–144). However, inhibiting the Btk pathway can be a double-edge sword during infection, since these kinases are also needed for an efficient immune response. Finding the balance to limit platelet activation without affecting the immune response and without increasing the risk of bleeding is the biggest challenge for developing new treatments to prevent or to limit thrombosis under infection-driven inflammatory conditions.

### Neutrophil Activation

Platelets express a wide range of classical immune receptors such as TLRs (145). Activation of TLR4 with LPS promotes binding of platelets to neutrophils and can promote NET formation (146). The involvement of platelets in promoting NETs has been described in *in vivo* models of sepsis and viral infection (133, 147). In an *E. coli*-induced sepsis model, NETs within the microvasculature could help trap bacteria, yet their induction also increased organ damage and promoted thrombosis through a NET-platelet-thrombin pathway (148). This is due to the many NET-derived elements that have pro-coagulant properties, including histones, DNA, and proteases that can activate coagulation through either the extrinsic or intrinsinc pathway (148). PAD4 ^−/−^ mice do not generate NETs after systemic bacterial exposure in the CLP model. Despite this, PAD4 ^−/−^ mice control the infection to the same level as wild-type, but experience lower levels of inflammation and thrombosis (149). Although PAD4 is essential for the development of NETs, it is dispensible for NET formation once the process has started (149). Thus, blocking PAD4 may be a prophylactic way to target NET development, but is unlikely to be a therapeutic route. To achieve this, it may be necessary to block the whole PAD pathway. Since increased free DNA from NETs contributes to vascular occlusion it means targeting NETs with DNases may be a route to protect against the harmful effects of NETosis (150). However, the timing of when to inhibit NETs is likely to be important. Administration of DNase during the early stages of CLP (2 h after surgery) is detrimental, resulting in enhanced levels of inflammatory cytokines. In contrast, delaying treatment with DNase to 4–6 h after puncture can improve the clinical score and bacteria control (151). STm infection can stimulate NETs *in vitro* and these NETs can trap bacteria (152). Nevertheless, the role of neutrophils and NETs in thrombosis after infection has not been described.

## Conclusion

Infection-mediated thrombosis links the inflammatory response induced in response to the pathogen with the coagulation system. Further research that uses longer-term infection models may help elucidate the full consequences of driving thrombosis and the wider mechanisms behind thrombus development. The numbers of models available are limited and may not fully represent the events experienced by humans during severe infections. Nevertheless, addressing the areas where we have a limited understanding of the drivers and consequences of infection-driven thrombosis will help us develop better strategies to prevent and treat thromboembolic complications without compromising the immune response against the pathogen or interfere with hemostasis.

## Author Contributions

NB-C contributed to the conception of the study, writing the manuscript, and generating the figures. MP-T contributed to writing the manuscript. MT proof-read the manuscript and contributed to the intellectual concepts. IH proof-read the manuscript. SW proof-read the manuscript and contributed to the intellectual concepts. AC co-wrote the manuscript and contributed to the intellectual concepts within the manuscript.

### Conflict of Interest

The authors declare that the research was conducted in the absence of any commercial or financial relationships that could be construed as a potential conflict of interest.
